# Investigation on Mode I Fracture Toughness of Woven Carbon Fiber-Reinforced Polymer Composites Incorporating Nanomaterials

**DOI:** 10.3390/polym12112512

**Published:** 2020-10-28

**Authors:** Gia Toai Truong, Hai Van Tran, Kyoung-Kyu Choi

**Affiliations:** School of Architecture, Soongsil University, 369 Sangdo-ro, Dongjak-gu, Seoul 06978, Korea; toai.truonggia@uah.edu.vn (G.T.T.); haivan993@gmail.com (H.V.T.)

**Keywords:** fracture toughness, fiber reinforced polymers (FRPs), nanomaterials, interface fiber angle, prediction model

## Abstract

This study experimentally investigated the effects of nanomaterials and interface fiber angle on the mode I fracture toughness of woven carbon fiber-reinforced polymer (CFRP) composites. Three different types of nanomaterials were used: COOH-functionalized short multi-walled carbon nanotubes (S-MWCNT-COOH), multi-walled carbon nanotubes (MWCNTs), and graphene nanoplatelets (GnPs). Double cantilever beam specimens were composed of 12 woven carbon fiber fabrics with/without 1 wt% nanomaterials, and were manufactured using the hand lay-up method. Furthermore, two different stacking sequence series were used; the first series comprised only on-axis carbon-fiber fabrics (0° or 90°), and the second series comprised both on- and off-axis carbon-fiber fabrics (0° or 90° and ±45°). The test results showed that adding S-MWCNT-COOH, MWCNTs, and GnPs significantly increased the mode I fracture toughness of the CFRP composites for both the stacking sequence series. Moreover, the specimens that used only on-axis carbon fiber fabrics exhibited higher fracture toughness values than those of the specimens that used on- and off-axis carbon fiber fabrics together. In addition, an empirical model was established to predict the fracture toughness of the CFRP composites with nanomaterials by using on- and off-axis carbon fiber fabrics together, and the prediction results showed a good agreement with the experimental results.

## 1. Introduction

Recently, the use of fiber reinforced polymer (FRP) composites has been widely increased in the field of civil and architectural engineering because of their high strength and stiffness. In addition, FRP composites have lower weights than those of other conventional construction materials. Due to the various advantages of FRP materials, they are globally used in various engineering fields [1,2,3,4]. However, from a mechanical viewpoint, FRP composites show complex failure modes such as matrix cracking, fiber rupture, and interlaminar delamination; therefore, their performance is limited [5,6,7,8,9,10]. Particularly, interlaminar delamination, which is usually attributed to shear or tensile cracking at the interfaces of FRP layers, is one of the most critical damage mechanisms. Additionally, adhesive materials exhibit brittle or quasi-brittle behaviors, and thus might result in the growth of delamination in composite materials [11]. Consequently, using tougher matrices might delay the onset of delamination. From previous studies [12,13,14,15,16,17,18,19], it was observed that using additive materials such as thermoplastic resins, rubber, and nanoparticles (or nanomaterials) into matrix could considerably enhance the toughness of the matrix, thereby improving the fracture toughness of FRP because of the increased interlaminar strength.

Numerous studies have been performed to investigate the delamination resistance (e.g., fracture toughness) of FRP composites using modified matrices. Ozdemir et al. [20] investigated the effects of rubber nanoparticles (carboxylic acrylonitrile butadiene rubber and acrylonitrile butadiene rubber) on the fracture toughness of carbon fiber-reinforced polymer (CFRP) composites with dicyandiamide-cured epoxy matrices. The test results obtained indicated that for the specimens using 16.7 wt% rubber nanoparticles, the mode I fracture toughness increased by approximately 250%. In the study by Carolan et al. [21], the fracture characteristics of CFRPs were investigated at two temperature levels of 20 °C and −80 °C; these characteristics were based on the matrices of an anhydride-cured epoxy resin and incorporated silica nanoparticles or polysiloxane core–shell rubber (CSR) as additives. Generally, at both the temperature levels, the use of epoxy resins by incorporating silica and CSR nanoparticles was observed to significantly improve the mode I fracture toughness of the CFRP composites. In addition, the CFRP composites using silica nanoparticles showed a higher toughness energy at −80 °C than that at 20 °C. However, using such rubber nanoparticles may cause the dispersion problem of the nanophased matrix during the fabrication process while coating the dry fiber fabrics surfaces [22].

In other research works [23,24,25], nanomaterials such as carbon nanotubes (CNTs) and carbon nanofibers were introduced to enhance the fracture toughness of polymer matrices. This is because the nanomaterials that have high stiffness and strength play a role as reinforcement materials, which leads to an enhancement in the mechanical performance of composites. Hamer et al. [26] investigated the interlaminar fracture toughness values (*G_Ic_* and *G_IIc_*) of CFRP laminates that comprised a hybrid resin with 5 wt% of multiwalled carbon nantotubes (MWCNTs). The test results indicated that the addition of MWCNTs to the epoxy resin could enhance not only mode I fracture toughness (*G_Ic_*) but also mode II fracture toughness (*G_IIc_*) of CFRP composites. Consequently, both *G_Ic_* and *G_IIc_* were improved by 25% and 20%, respectively in comparison with non-interleaved CFRPs. This improvement was mainly attributed to the effectiveness of MWCNTs in arresting crack propagation in the interlayer. In addition, Wicks et al. [27] showed that using long aligned CNTs could significantly improve the fracture toughness in steady state, which was in contrast to the results obtained with using short CNTs. This is because long CNTs result in rough fracture surface of epoxy resin and, therefore, dissipate more energy during the pull-out process. Moreover, various studies [28,29,30,31,32,33,34,35] indicated that the mode I fracture toughness of CFRP composites was also affected by the type, amount, length, and density of CNTs, interface fiber angle, and stacking sequences of carbon fiber fabrics. However, excessive amounts of CNTs could not continuously improve the fracture toughness of CFRP composites because of poor dispersion or the agglomeration of CNTs [36].

In this study, the mode I fracture toughness of woven CFRP composites incorporating nanomaterials was investigated using double cantilever beam (DCB) specimens. Three different nanomaterial types were used, namely: 1 wt% MWCNTs, 1 wt% graphene nanoplatelets (GnPs), and 1 wt% COOH-functionalized short MWCNTs (S-MWCNT-COOH). The test specimens comprised 12 carbon fiber fabrics. Two different stacking sequences series were used: the first series comprised only on-axis carbon fiber fabrics (0° or 90°), while the second series comprised both on- and off-axis carbon fiber fabrics (0° or 90° and ±45°) with an alternating arrangement. An empirical model was proposed for predicting the mode I fracture toughness of CFRP composites incorporating nanomaterials with both on- and off-axis carbon fiber fabrics.

## 2. Experimental Program

### 2.1. Material Properties

**Figure 1:**
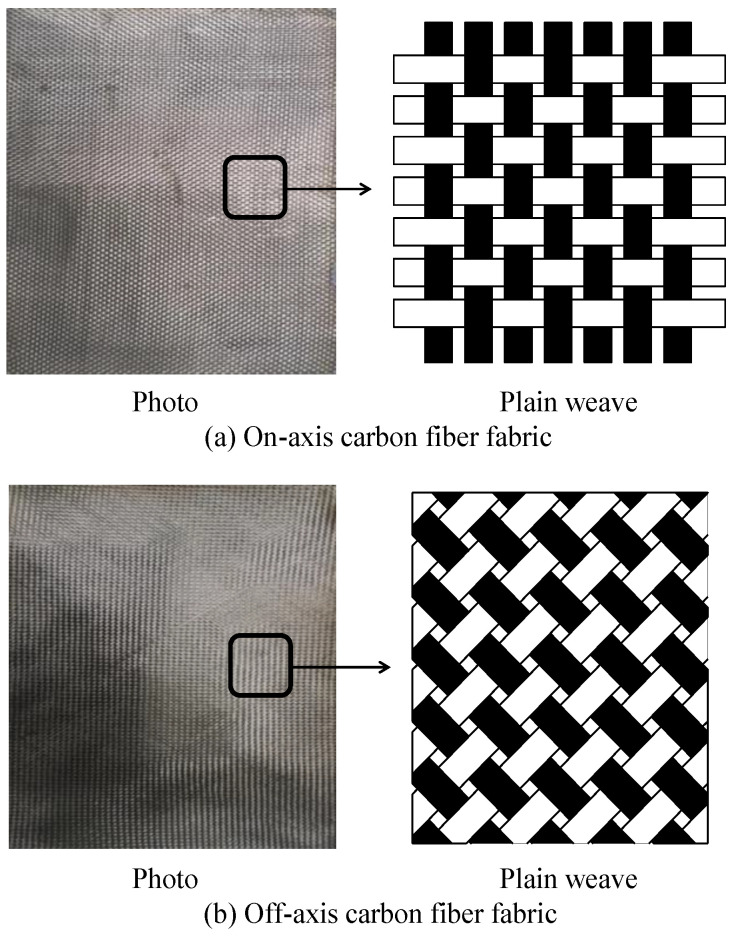
Carbon fiber fabrics used in this study: (**a**) On-axis carbon fiber fabric and (**b**) Off-axis carbon fiber fabric.

In this study, 0.21-mm thick woven carbon fiber fabrics that consisted of 3k filaments were used as reinforcement. The average density of the carbon fiber fabrics was 1.76 g/cm^3^. The tensile strength, elastic modulus, and ultimate tensile strain of the carbon-fiber fabrics were 3530 MPa, 230,000 MPa, and 1.5%, respectively, as provided by the manufacturer. Figure 1 depicts the carbon fiber fabrics used in this study. In the figure, the carbon fiber filaments of both on- and off-axis carbon fiber fabrics have an orientation of 0° (or 90°) and ±45° with respect to the longitudinal direction, respectively.

Three types of nanomaterials, namely, MWCNTs, GnPs, and S-MWCNT-COOH, were used as nanofillers. The geometries and properties of these nanomaterials are presented in Table 1. Generally, these nanomaterials were manufactured with a purity of more than 95 wt%. In addition, each nanomaterial type was different in term of length, diameter, and surface area. The epoxy resin and the corresponding polyamidoamine hardener were used as matrix. Both the epoxy resin and hardener had low viscosities ranging from 800 to 1600 cps and from 500 to 1000 cps, respectively, at 25 °C. The stoichiometric ratio between the epoxy resin and hardener was 100:55 by weight. More details of the epoxy resin and hardener were presented in the study by Truong et al. [37].

### 2.2. Manufacture of the Test Specimen

As mentioned above, in this study, three different types of nanomaterials, namely, MWCNTs, GnPs, and S-MWCNT-COOH, were used. While mixing the nanomaterials into epoxy, many accidents might occur such as agglomeration, out of alignment, poor dispersion, and damage of nanomaterials, thereby reducing the mechanical properties of CFRP composites. For ensuring uniform dispersion of the nanomaterials in the epoxy resin, using nanomaterials in appropriate amount is one of the key issues. As reported in previous studies [28,36], using a small amount of nanomaterials can avoid agglomeration and maintain uniform dispersion of nanomaterials in epoxy resin. In this study, considering the surface area and aspect ratios, 1 wt% (by weight) of epoxy resin/hardener mixture of each of the nanomaterials of MWCNTs, GnPs, and S-MWCNT-COOH were used. Prior to fabricating the CFRP composites, the epoxy resin was filled with nanomaterials by dispersing nanomaterials on the epoxy resin by using a sonicator. The mixing procedure of these epoxy–nanocomposite mixtures was presented in the study by Truong et al. [37].

**Figure 2:**
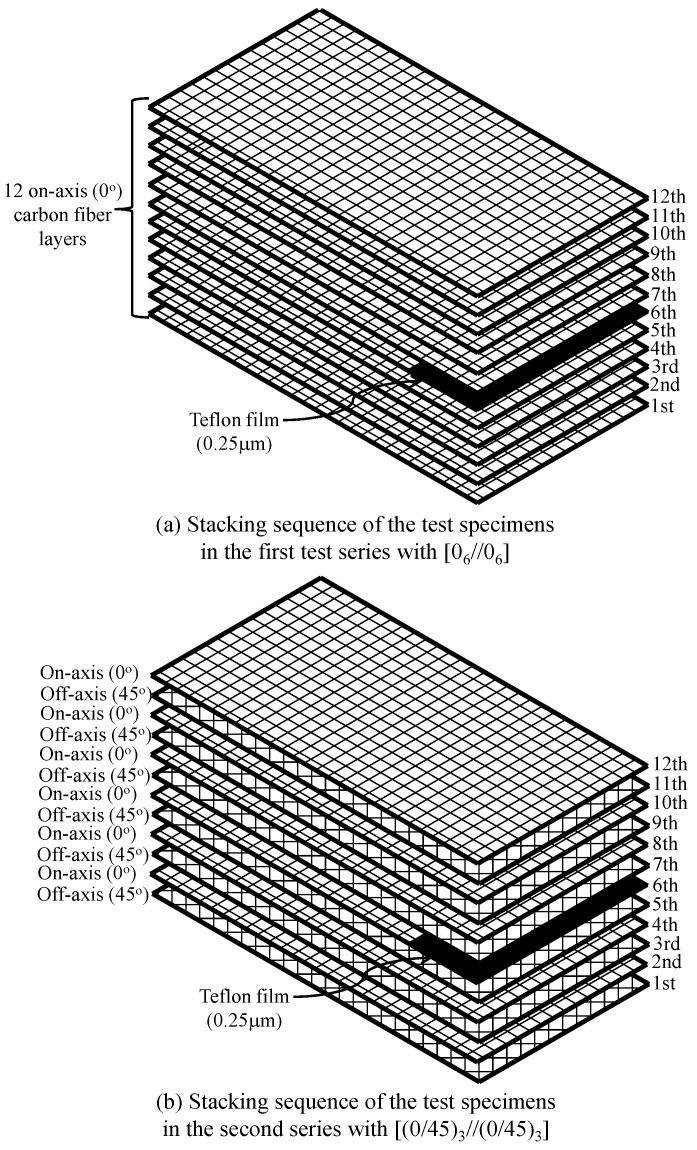
Stacking sequences of the test specimens used in this study: (**a**) Stacking sequence of the test specimens in the first series with [0_6_//0_6_] and (**b**) stacking sequence of the test specimens in the second series with [(0/45)_3_//(0/45)_3_].

Various methods can be applied to fabricate FRP composites [38,39]. In this study, for easy application, the hand lay-up method was used to fabricate CFRP composite plates. Twelve carbon fiber fabrics with 300 mm length and 150 mm width were used to fabricate each CFRP composite plate. The carbon fiber fabrics were coated using the epoxy–nanocomposite mixture on two surfaces by using a brush and then placed on acrylic plates, which were used as mold, according to the design configurations. Notably, before placing the carbon fiber fabrics, the surfaces of the acrylic plates were coated using a releasing agent for ensuring easy release of the composite specimens. In addition, at one edge of the composites, a 25-μm-thick Teflon film was inserted into the mid-section located between 6th and 7th carbon fiber layers as depicted in Figure 2. The CFRP composites were then cured in the room temperature (approximately 20 °C) for 7 days under an appropriate pressure of 20 kPa. Using the fabricated CFRP composite plates, DCB specimens were prepared by cutting the composite plates for performing fracture tests (Bosch, Stuttgart, Germany). Five duplicates of the test specimens were made for each parameter. However, one or two duplicates of GnPs-CFRP0-0 or MWCNT-CFRP0-45 were broken after manufacture. Therefore, in total, 37 test specimens were tested.

Two different series of DCB specimens were manufactured and tested. The first series had only on-axis carbon fiber fabrics, whose stacking sequence (or layups) was denoted by [0_6_//0_6_]. In the second series, six on-axis and six off-axis carbon fiber layers were alternately arranged in the order of 0° (or 90°) and ±45°, and the stacking sequence was denoted by [(0/45)_3_//(0/45)_3_]. The numbers “0” and “45” denote the orientations of adjacent carbon fiber layers; the subscripts “6” and “3” represent the repeat times of the same order. The symbol “//” provides the position of the initial crack of the test specimens. Figure 2 depicts the stacking sequences of the test specimens in this study. According to such stacking sequences, at the mid-plane of the test specimens (cracking surface), in the first series, both 0° (or 90°) carbon fiber fabrics were used and denoted by 0//0 interface fiber angle, while in the second series, 0° (or 90°) and ±45° carbon fiber fabrics were used and denoted by 0//45 interface fiber angle.

To distinguish these DCB test specimens from one another, they were named CFRP0-0, S-MWCNT-COOH-CFRP0-0, MWCNT-CFRP0-0, and GnPs-CFRP0-0 for the stacking sequence of [0_6_//0_6_], and CFRP0-45, S-MWCNT-COOH-CFRP0-45, MWCNT-CFRP0-45, and GnPs-CFRP0-45 for the stacking sequence of [(0/45)_3_//(0/45)_3_]. Among them, CFRP0-0 and CFRP0-45 were the control specimens without nanomaterials. The numbers, namely, 0-0 and 0-45, after the term “CFRP” indicate the interface fiber angles corresponding to the stacking sequences used to fabricate the DCB test specimens.

**Figure 3:**
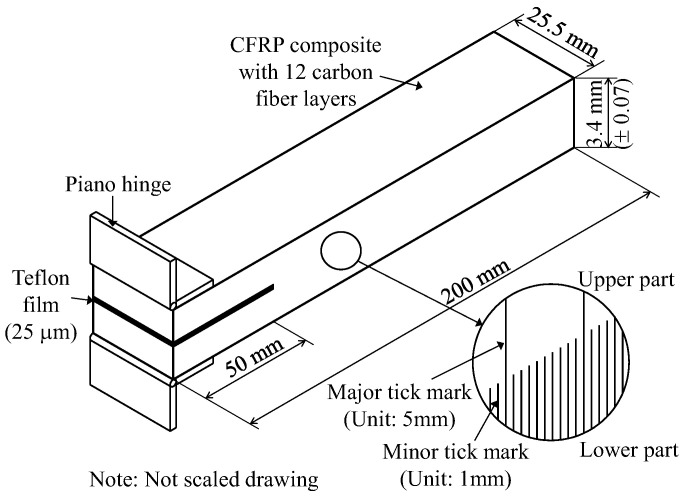
Details of the DCB test specimens.

Figure 3 depicts the details of the DCB specimens used in this study; the configurations of the specimens were based on ASTM D5528-13 [40]. In the figure, a DCB specimen was designed to have length of 200 mm, width of 25.5 mm, and pre-delamination length of 50 mm. While evaluating the fracture toughness of the DCB specimens, the width and thickness directly measured from the test were used. In the case of the first series with the stacking sequence of [0_6_//0_6_], the average thicknesses for the specimens CFRP0-0, S-MWCNT-COOH-CFRP0-0, MWCNT-CFRP0-0, and GnPs-CFRP0-0 were 3.32, 3.42, 3.52, and 3.38 mm, respectively. In addition, in the case of the second series with the stacking sequence of [(0/45)_3_//(0/45)_3_], the average thicknesses of the specimens CFRP0-45, S-MWCNT-COOH-CFRP0-45, MWCNT-CFRP0-45, and GnPs-CFRP0-45 were 3.32, 4.42, 3.50, and 3.46 mm, respectively. It can be seen that upon the addition of nanomaterials, the thickness of the DCB composites slightly increased compared with those of the control specimens. Notably, the maximum increase in the thickness was only 5.90% for the MWCNT-CFRP0-0 specimen; such a slight variation in thickness did not considerably affect the fracture toughness of the composites because the crack grew only along the mid-plane of the specimens. In addition, in the pre-delamination zone, two steel hinge end tabs were attached to the surfaces of the DCB specimens, for applying load. The sides of the specimens were also painted white and marked using a millimeter scale to aid in the visual detection of the crack (or delamination) growth.

### 2.3. Carbon-Fiber Volume Fraction

To accurately examine the characteristics of the DCB test specimens, the carbon fiber volume fractions were evaluated as follows [41]:

(1) $$v_{f}=\frac{n}{h}\frac{A_{w}}{\rho_{f}}$$

where *n* is the number of plies, $A_{w}$ (= 198 g/m^2^) is the fiber areal weight, $\rho_{f}$ (= 1.76 g/cm^3^) is the fiber density, and *h* is the specimen thickness. In the case of the first series, the carbon fiber volume fractions of CFRP0-0, S-MWCNT-COOH-CFRP0-0, MWCNT-CFRP0-0, and GnPs-CFRP0-0 were 40.61%, 39.31%, 38.35%, and 39.94%, respectively. In the case of the second series, the carbon fiber volume fractions of CFRP0-45, S-MWCNT-COOH-CFRP0-45, MWCNT-CFRP0-45, and GnPs-CFRP0-45 were 40.63%, 39.50%, 38.54%, and 39.07%, respectively. Generally, the carbon fiber volume fractions of the DCB test specimens slightly varied by approximately 2.5%. The slight variation in the carbon fiber volume fractions was attributed to the different thicknesses of the test specimens, as mentioned above.

### 2.4. Test Setup for Mode I Fracture Toughness

**Figure 4:**
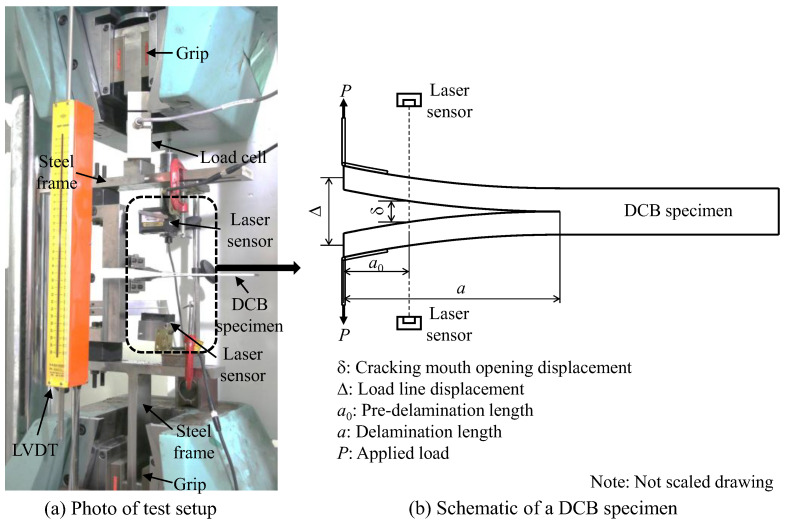
DCB test setup for investigating the mode I fracture toughness: (**a**) Photo of test setup and (**b**) Schematic of a DCB specimen.

The mode I fracture test of the DCB specimens was performed using a universal testing machine (UTM, Kyoungsung Testing Machine Co., Ansan, Korea) in the displacement control condition at the constant cross-head speed of 1 mm/min. Figure 4 depicts the test setup for the DCB testing. To measure the applied load during the testing, a load cell with the capacity of 5 kN was used. In addition, the load line displacements of the test specimens were measured using two different methods based on linear variable differential transformer (LVDT, Tokyo Measuring Instruments Laboratory Co., Tokyo, Japan) and the cross-head movement, respectively. Notably, in most previous studies, the load line displacement was directly obtained from the cross-head movement [30,42,43]. It was observed that the displacement values obtained from each LVDT and cross-head movement were almost the same. Therefore, hereafter, in the investigation of the fracture toughness, the LVDT data have been used. In this study, the LVDT was not directly mounted on the DCB specimens but on the UTM, to record the displacement.

To calculate the fracture toughness, the delamination length (*a*), applied load (*P*), and displacement (Δ) of loading point should be recorded. A high-resolution digital camera (iX Cameras Ltd, Essex, UK) was used to take the photograph of the side of the DCB specimens, to record the crack propagation. The crack mouth opening displacement (CMOD), *δ*, was also determined using two laser displacement sensors (Keyence, Itasca, IL, USA), which were, respectively, mounted above and below the steel frames so that the laser ray could coincide with the end point of the pre-crack length ($a_{0}$ = 50 mm).

The modified compliance calibration (MCC) method was used to characterize the interlaminar fracture toughness, $G_{Ic}$ (in kJ/m^2^), of the DCB specimens as follows:

(2) $$G_{Ic}=\frac{3P^{2}C^{2/3}}{2A_{1}bh}$$

where *C* (= Δ/P) is the compliance, *b* is the specimen width, and *A*_1_ is a data reduction factor obtained from the experimental results. In the MCC method, *A*_1_ is determined as a coefficient that explains the linear relationship between the normalized delamination length (*a/h*) by using the specimen thickness and the cube root of compliance (*C*^1/3^).

## 3. Test Results and Discussions

### 3.1. Load–Displacement Curve

**Figure 5:**
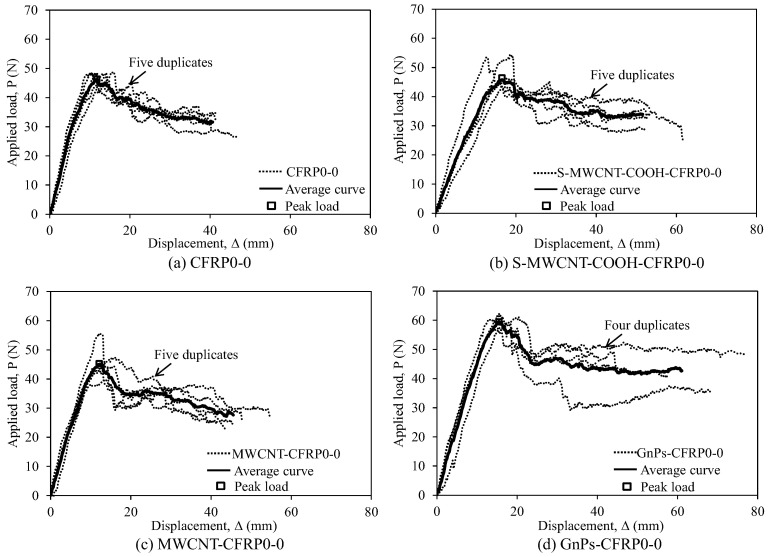
Load–displacement relationship for the test specimens in the test series with the stacking sequence of [0_6_//0_6_]: (**a**) CFRP0-0, (**b**) S-MWCNT-COOH-CFRP0-0, (**c**) MWCNT-CFRP0-0, and (**d**) GnPs-CFRP0-0.

**Figure 6:**
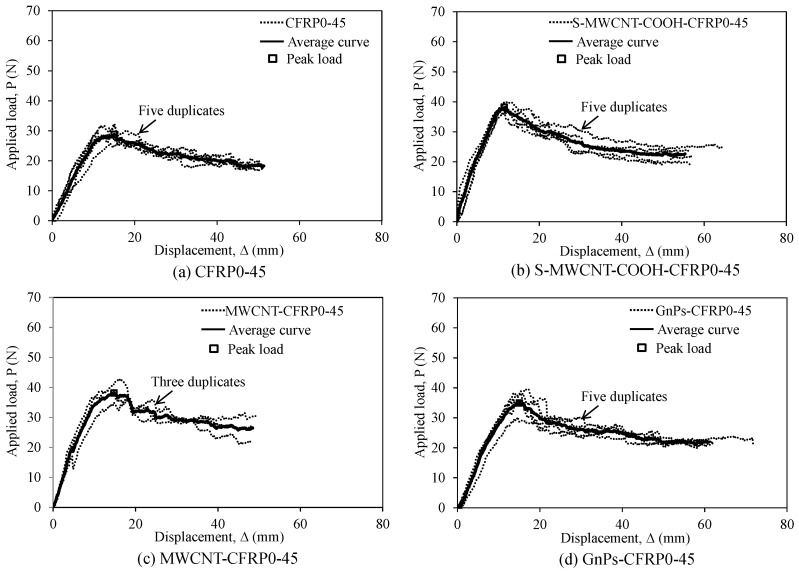
Load–displacement relationship for the test specimens in the test series with the stacking sequence of [(0/45)_3_//(0/45)_3_]: (**a**) CFRP0-45, (**b**) S-MWCNT-COOH-CFRP0-45, (**c**) MWCNT-CFRP0-45, and (**d**) GnPs-CFRP0-45.

Figure 5 and Figure 6 depict the applied load–displacement responses of the test specimens in the first and second series with the stacking sequences of [0_6_//0_6_] and [(0/45)_3_//(0/45)_3_], respectively. In addition, the average applied load–displacement curves of these test specimens are depicted. In the case of the first series (see Figure 5), the test specimens exhibited almost linear behavior in the early ascending branch up to the inflection point wherein delamination begins to develop at the mid-plane of the DCB specimens. Following the linear part, the slope of the load–displacement curve begins to decrease because of crack propagation; however, the load-carrying capacity of the test specimens continues to increase up to a peak load because of fiber bridging. Immediately upon reaching the peak load, in the early descending branch, a visible crack propagation occurred, and the applied load showed a sudden partial decrease. Subsequently, in all the DCB specimens, the applied load gradually decreased with a slight fluctuation. In the case of the second series (see Figure 6), similar behaviors as in the case of the first series were observed.

**Figure 7:**
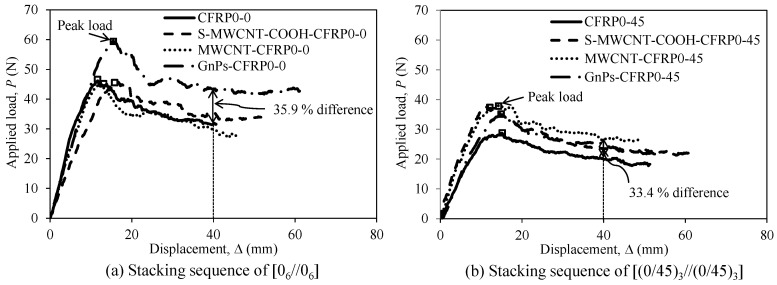
Average load–displacement relationship of the DCB test specimens incorporating nanomaterials: (**a**) Stacking sequence of [0_6_//0_6_] and (**b**) stacking sequence of [(0/45)_3_//(0/45)_3_].

In Figure 7, we compare the average applied load–displacement relationships of the DCB test specimens: control specimens, namely, CFRP0-0 and CFRP0-45, and other specimens incorporating 1 wt% of each S-MWCNT-COOH, MWCNTs, and GnPs. The slope of the linear part is strongly related to the flexural rigidity of each arm of the specimens. The flexural rigidity values of the test specimens were evaluated at the point wherein the applied load was approximately 20% of the peak load in the ascending branch of the curves. In the first series with the stacking sequence of [0_6_//0_6_] (see Figure 7a and Table 2), the nanomaterials at the mid-plane of the DCB specimens did not considerably increase the flexural rigidity of the test specimens before crack propagation; the flexural rigidities of specimens CFRP0-0, MWCNT-CFRP0-0, and GnPs-CFRP0-0 were almost the same at approximately 5.06–5.39 N/mm, while that of S-MWCNT-COOH-CFRP0-0 was relatively low at 3.79 N/mm. Considering the peak load, GnPs-CFRP0-0 displayed approximately 29.5% higher value (60.58 N) than those displayed by the other specimens including CFRP0-0. Regarding the residual load, at a displacement value of 40 mm, GnPs-CFRP0-0 displayed 35.9% higher value than those displayed by other specimens, which had almost the same residual load (see Table 2).

In the second series with the stacking sequence of [(0/45)_3_//(0/45)_3_] (see Figure 7b and Table 2), each nanomaterial had a different effect on not only the flexural rigidity but also the peak and residual loads of the DCB specimens. The flexural rigidities of S-MWCNT-CFRP0-45 and MWCNT-CFRP0-45 were 5.53 and 4.10 N/mm, respectively, which were significantly higher than those of CFRP0-45 and GnPs-CFRP0-45. In addition, using nanomaterials increased the peak and residual loads of the test specimens, as evident from Table 2. Among the test specimens, MWCNT-CFRP0-45 displayed the maximum increase by 31.9% for the peak load and 33.4% for the residual load, compared with those of CFRP0-45.

**Figure 8:**
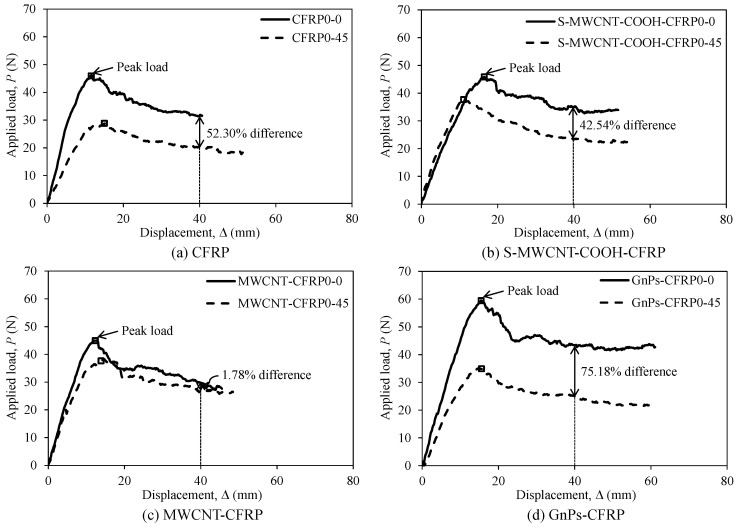
Average load–displacement relationship of the DCB test specimens displaying the effect of stacking sequence: (**a**) CFRP, (**b**) S-MWCNT-COOH-CFRP, (**c**) MWCNT-CFRP, and (**d**) GnPs-CFRP.

The effect of the interface fiber angles on the applied load–displacement relationships of the test specimens was investigated, and the comparisons are depicted in Figure 8. As depicted in the figure, generally, the flexural rigidity of each specimen in the first series with the stacking sequence of [0_6_//0_6_] was considerably higher than that of the corresponding specimen in the second series with the stacking sequence of [(0/45)_3_//(0/45)_3_], except for S-MWCNT-COOH-CFRP (see Figure 8b). In addition, the peak load of each specimen in the first series was approximately 16.13–68.58% higher than that of the corresponding specimen in the second series (see Table 2). Considering the residual load, each specimen in the first series produced higher value than that produced by the corresponding specimen in the second series, while MWCNT-CFRP0-45 exhibited almost the same value as that of MWCNT-CFRP0-0.

**Figure 9:**
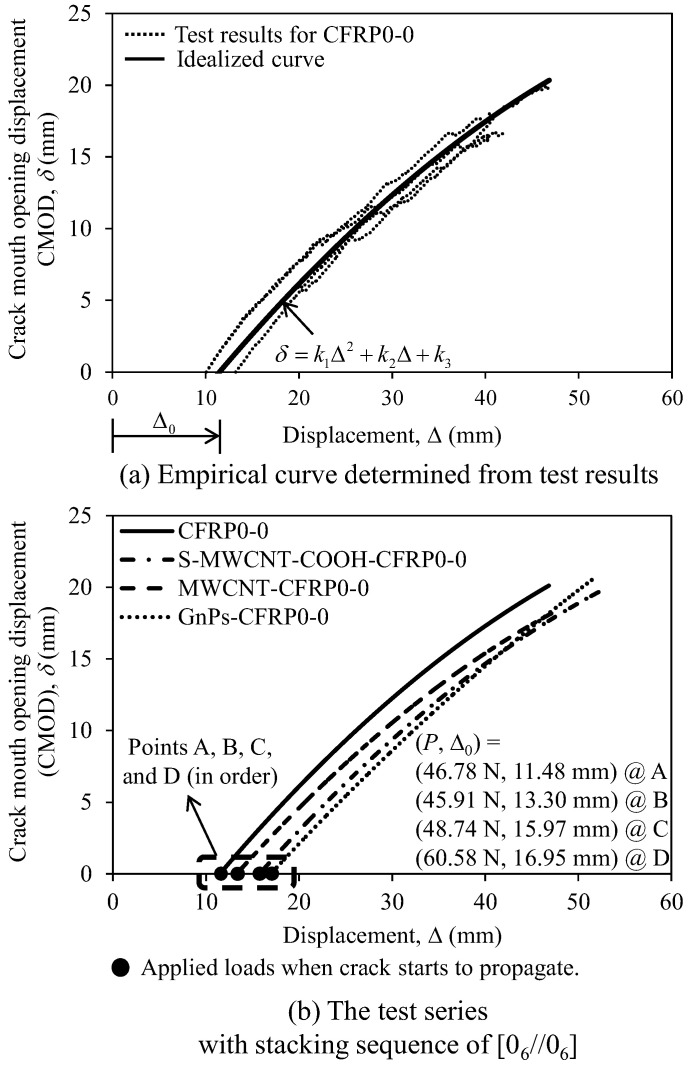
Crack mouth opening displacement (CMOD) versus load line displacement: (**a**) Empirical curve determined from the test results and (**b**) The test series with stacking sequence of [0_6_//0_6_].

A typical CMOD, *δ*, measured using laser displacement sensors versus the load line displacement of CFRP0-0 specimen is depicted in Figure 9a. In the figure, the CMOD versus load line displacement curve is slightly nonlinearity. Based on the experimental data, an empirical curve of CMOD (*δ*) versus the load line displacement was proposed as follows:

(3) $$\delta =k_{1}\Delta^{2}+k_{2}\Delta +k_{3}$$

where $k_{1}$, $k_{2}$, and $k_{3}$ are factors for the best fit between test results and prediction, and their values are presented in Table 3. In Figure 9 and Table 3, the empirical curves and the load line displacement ($\Delta_{0}$) of the test specimens are presented. Notably, the crack begins to propagate at the displacement of $\Delta_{0}$.

Figure 9b depicts the load line displacement versus CMOD curves of the test specimens in the first series. The figure indicates that the specimens using nanomaterials displayed higher $\Delta_{0}$ than that (11.48 mm) of the control specimen CFRP0-0. Particularly, GnPs-CFRP0-0 with 1 wt% GnPs exhibited the highest $\Delta_{0}$ (16.95 mm) at the peak load (60.58 N), which were considerably higher than those of CFRP0-0, thereby implying that nanomaterials at the mid-plane of the specimens could delay the crack development.

### 3.2. Data Reduction Factor (A_1_)

**Figure 10:**
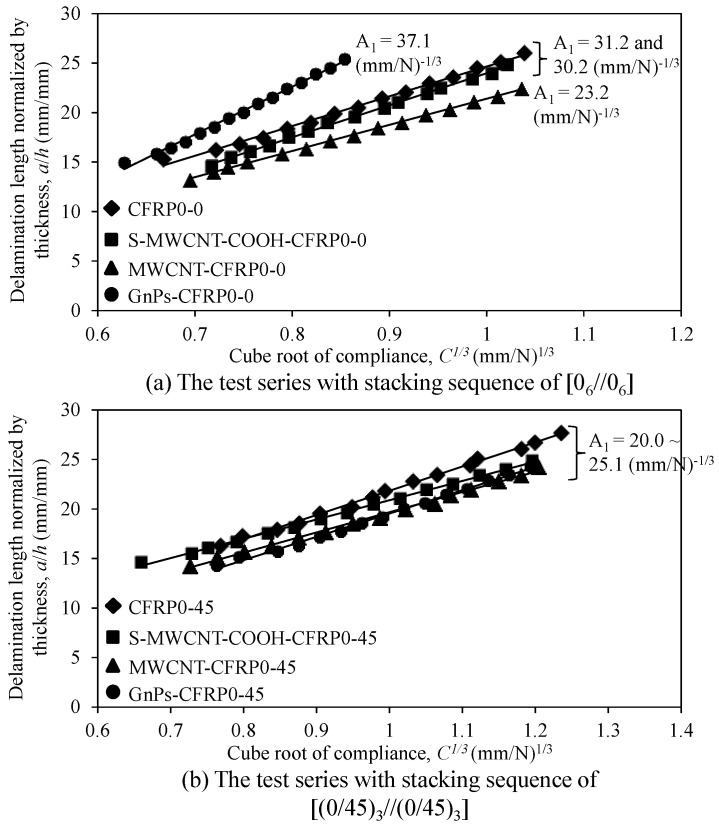
Delamination length normalized by thickness versus cube root of the compliance of DCB specimens: (**a**) The test series with stacking sequence of [0_6_//0_6_] and (**b**) the test series with stacking sequence of [(0/45)_3_//(0/45)_3_].

To evaluate the fracture toughness of the DCB specimens according to the MCC method (see Equation (2)), the data reduction factor (*A*_1_) must be determined. Figure 10 depicts the curves showing the relationship between the delamination length (*a/h*) normalized by specimen thickness and the cubic root (*C*^1/3^) of the compliance for the DCB test specimens. As depicted in the figure, the data reduction factor varies for different nanomaterials and stacking sequences. In Figure 10a, the slope (*A*_1_ = 37.1 (mm/N)^−1/3^) of the linear plot of the curve for GnPs-CFRP0-0 is higher than those of the other specimens; CFRP0-0 and S-MWCNT-COOH-CFRP0-0 show almost similar slopes of 31.2 and 30.2 (mm/N)^−1/3^, respectively, and MWCNT-CFRP0-0 shows the lowest value of 23.2 (mm/N)^−1/3^. Meanwhile, in the second series with a stacking sequence of [(0/45)^3^//(0/45)^3^], as depicted in Figure 10b, the slopes did not show considerable difference; CFRP0-45 and MWCNT-CFRP0-45 showed a constant slope value of 25.0 (mm/N)^−1/3^, and S-MWCNT-COOH-CFRP0-45 and GnPs-CFRP0-45 showed a constant slope value of 20.0 (mm/N)^−1/3^. The average values of *A_1_* obtained from the test results are summarized in Table 4. Based on the limited test data obtained in this study, the data reduction factor *A_1_* was proposed and presented in Table 4.

### 3.3. Effect of Nanomaterials on Mode I Fracture Toughness

**Figure 11:**
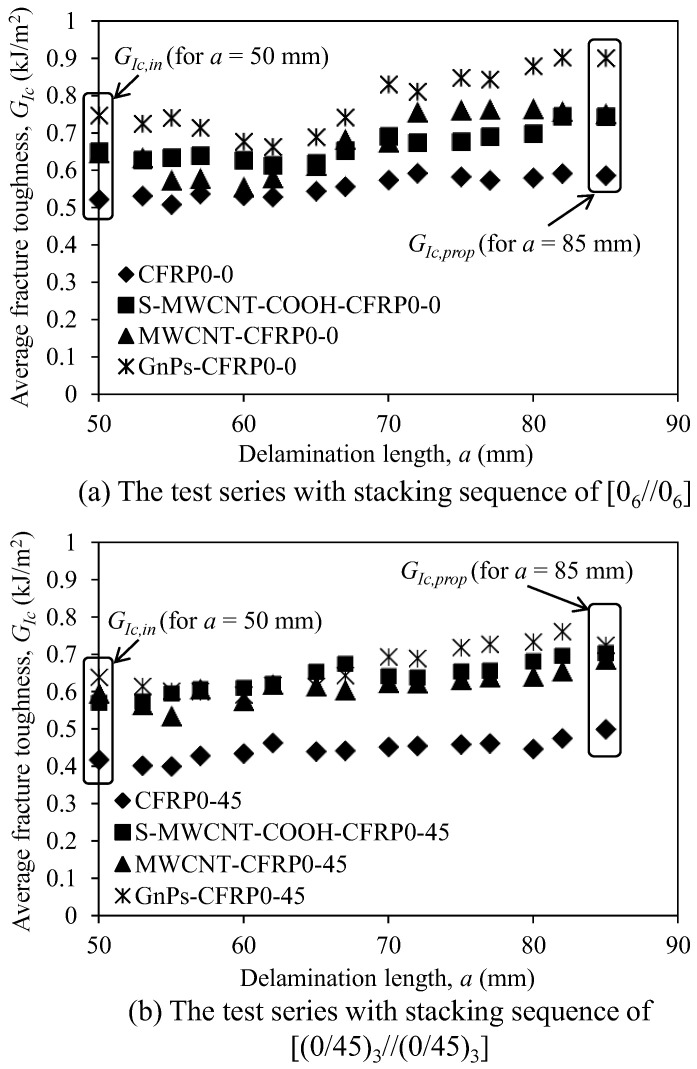
Fracture toughness versus crack delamination length of the DCB test specimens: (**a**) The test series with stacking sequence of [0_6_//0_6_] and (**b**) the test series with stacking sequence of [(0/45)_3_//(0/45)_3_].

Figure 11 and Table 5 present the average mode I fracture toughness ($G_{Ic}$) of the test specimens with respect to the delamination length (or crack growth). Notably, $G_{Ic}$ is evaluated using Equation (2) with the help of the data reduction factor *A*_1_ obtained from the test results. As depicted in the figure, the fracture toughnesses of all the specimens slightly increased with increase in the delamination length (*a*); this trend was also observed in the studies by Srivastava et al. [28] and Rehan et al. [43]. From Figure 11, it can be seen that adding nanomaterials to epoxy significantly increased the fracture toughness of the CFRP composites compared with that of pure CFRP.

**Figure 12:**
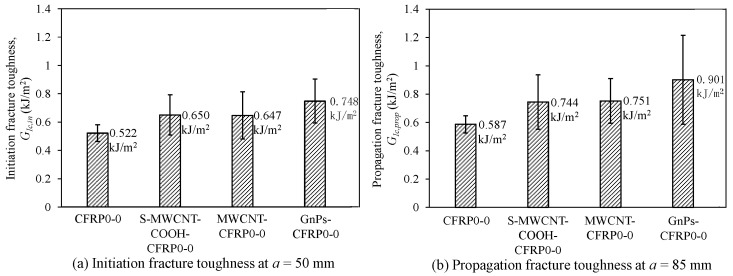
Effect of nanomaterials on (**a**) initiation fracture toughness at *a* = 50 mm and (**b**) propagation fracture toughness at *a* = 85 mm of the DCB specimens for the stacking sequence of [0_6_//0_6_].

In Figure 12, we compare the mode I fracture toughness of the CFRP composites incorporating nanomaterials with that of pure CFRP composites in the first series. Considering the initiation fracture toughness ($G_{Ic,in}$) at *a* = *a*_0_ (= 50 mm), as depicted in Figure 12a, using GnPs as nanofiller most effectively improved the initiation fracture toughness of the CFRP composites. The initiation fracture toughness of GnPs-CFRP0-0 was 43.2% higher than that of the control specimen CFRP0-0; meanwhile, S-MWCNT-COOH-CFRP0-0 and MWCNT-CFRP0-0 displayed approximately 24% higher values than that of CFRP0-0. Considering the propagation fracture toughness ($G_{Ic,prop}$) at a = 85 mm (see Figure 12b), generally, the trend is similar to that of initiation fracture toughness ($G_{Ic,in}$). As depicted in Figure 12b, GnPs-CFRP0-0 exhibited 53.6% higher propagation fracture toughness than that of CFRP0-0; meanwhile, S-MWCNT-COOH-CFRP0-0 and MWCNT-CFRP0-0 displayed approximately 27% higher values than that of CFRP0-0. In addition, the test results showed that the fracture toughness of S-MWCNT-COOH-CFRP0-0 and MWCNT-CFRP0-0 did not show significant difference. This is because the magnitude of fracture toughness of CFRP composites incorporating CNTs is dependent on the failure mechanism, which depends on not only the CNT length, but also the critical length of CNTs embedded in the epoxy matrix [44]. Additionally, since the values of the other parameters (such as CNT length, CNT diameter, interfacial shear strength between CNTs and epoxy resin, and chemical reaction between functionalized CNTs and epoxy resin [45]) are wide in range, in some cases, the fracture toughness might not be clearly different. Statistical analysis using two independent sample *t*-test with a statistical significant level (*α*) of 10% was used to analyze the different between two groups of experimental data regarding to *G_Ic,in_* and *G_Ic,prop_* [46,47]. The statistical analysis results indicated that the group of GnPs-CFRP0-0 showed significant difference in *G_Ic,in_* at the 90% confidence level compared to the control group of CFRP0-0. Meanwhile, S-MWCNT-COOH-CFRP0-0 and MWCNT-CFRP0-0 show a slight difference in *G_Ic,in_* compared to CFRP0-0. Similarly, for the propagation fracture toughness, all groups showed a slight difference in *G_Ic,prop_* at 90% confident level compared to CFRP0-0.

**Figure 13:**
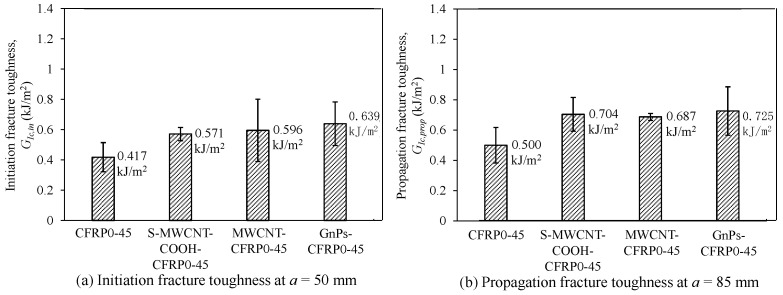
Effect of nanomaterials on (**a**) initiation fracture toughness at *a* = 50 mm and (**b**) propagation fracture toughness at *a* = 85 mm of the DCB specimens for the stacking sequence of [(0/45)_3_//(0/45)_3_].

In the second series (see Figure 13), the specimens that used GnPs as nanofiller showed the higher initiation and propagation fracture toughness than those of the control specimen CFRP0-45. However, the difference of the fracture toughness between GnPs-CFRP0-45 and the other test specimens with other nanofillers was not significant. In addition, similar to the test specimens with stacking sequence of [0_6_//0_6_], the fracture toughness of S-MWCNT-COOH-CFRP0-45 and MWCNT-CFRP0-45 did not show considerable difference. Based on the statistical analysis, it was found that the group of S-MWCNT-COOH-CFRP0-45 showed significant difference in *G_Ic,in_* at the 90% confidence level compared to the control group of CFRP0-45. Meanwhile, MWCNT-CFRP0-45 and GnPs-CFRP0-45 show a slight difference in *G_Ic,in_* compared to CFRP0-45. For the propagation fracture toughness, MWCNT- CFRP0-45 showed significant difference in *G_Ic,prop_* at the 90% confidence level compared to CFRP0-45. In contrast, S-MWCNT-CFRP0-45 and GnPs-CFRP0-45 showed a slight difference in *G_Ic,prop_* compared to CFRP0-45.

**Figure 14:**
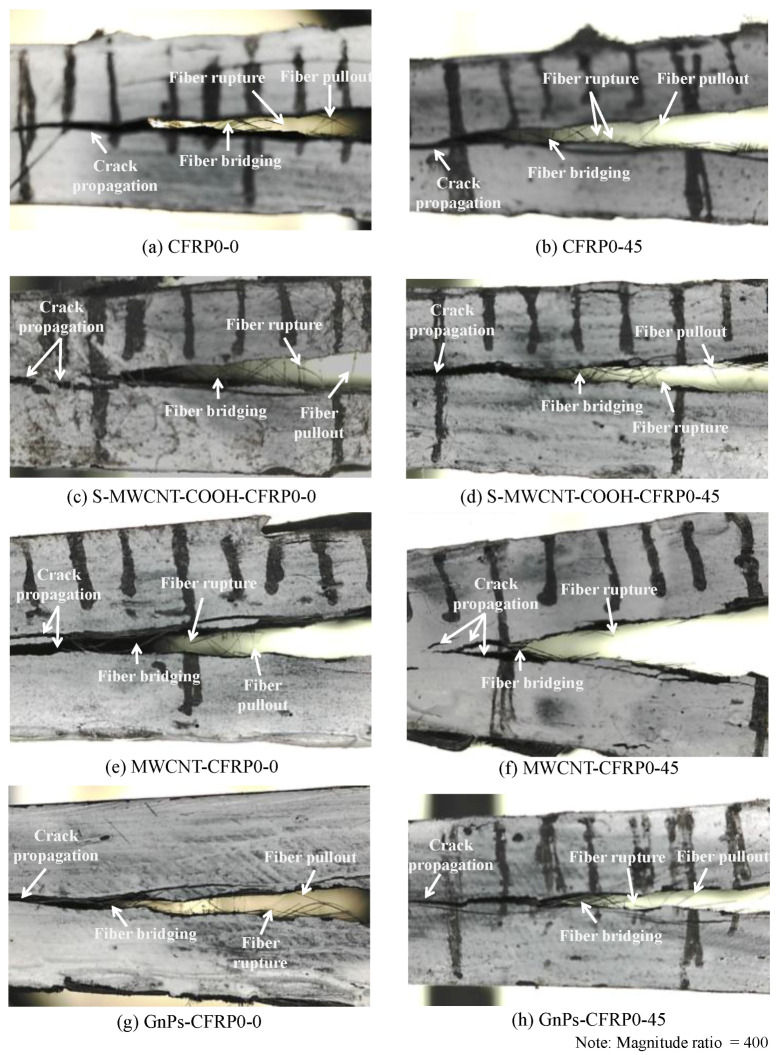
Fibers around the delamination zone of the CFRP composites: (**a**) CFRP0-0, (**b**) CFRP0-45, (**c**) S-MWCNT-COOH-CFRP0-0, (**d**) S-MWCNT-COOH-CFRP0-45, (**e**) MWCNT-CFRP0-0, (**f**) MWCNT-CFRP0-45, (**g**) GnPs-CFRP0-0, and (**h**) GnPs-CFRP0-45.

Figure 14 depicts the fiber around the mid-plane of the test specimens. It is evident that upon adding S-MWCNT-COOH, more fibers were placed, and multiple crack planes formed around the mid-plane of the test specimens, which might have contributed to bridging and the pull-out mechanism. According to Borowski et al. [48], this phenomenon could have resulted in more driving force and energy dissipation, thereby facilitating further crack propagation. Moreover, Davis and Whelan [49] and Li et al. [50] also indicated that CFRP composites containing nanomaterials might display rougher fracture surfaces at the mid-plane because of the toughening effect in the matrix compared with those of CFRP composites without nanomaterials; generally, a similar trend was observed in other DCB specimens incorporating nanomaterials.

### 3.4. Effect of Interface Fiber Angle

**Figure 15:**
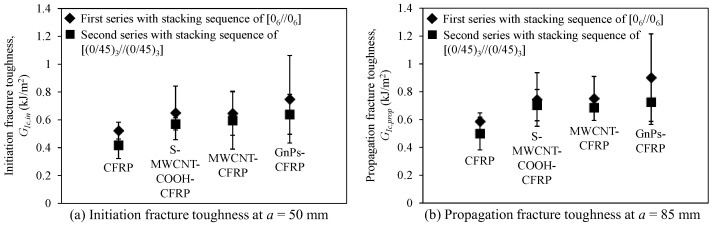
Effect of interface fiber angle on (**a**) initiation fracture toughness at *a* = 50 mm and (**b**) propagation fracture toughness at *a* = 85 mm of the DCB specimens.

In Figure 15, we draw comparisons among the fracture toughnesses of the DCB specimens with the interface fiber angles of 0//0 and 0//45 in the first and second series, respectively. In the figure, generally, in term of average, both initiation and propagation fracture toughnesses of the composites with the interface fiber angle of 0//0 were higher than those of the composites with the interface fiber angle of 0//45; this trend is the same as those observed in the studies by Rehan et al. [43] and Kharratzadeh et al. [51] using the pre-impregnated carbon epoxy and woven glass fibers. In Figure 15a, the difference of initiation fracture toughness between two different stacking sequences was approximately 7.9–20.0%. Gong et al. [52] and Rehan et al. [43] observed that carbon fiber layers with the fiber angle of 0//45 near the crack plane might cause more potential damage at the crack tip because of the increase in the microcracks in the epoxy resin within carbon fiber layers, thereby decreasing the initiation fracture toughness of the composites. The propagation fracture toughness of the DCB specimens displayed almost the same trend as that displayed by the initiation fracture toughness (see Figure 15b). The specimens using the first stacking sequence exhibited higher propagation fracture toughness of 5.7–24.3% than that of the corresponding specimens using the second stacking sequence. However, according to the *t*-test statistical analysis, at a confident level of 90%, the group of the test specimens with interface fiber angle of 0//0 was not clearly different in *G_Ic,in_* and *G_Ic,prop_* compared to the group of the test specimens with interface fiber angle of 0//45. Thus, further investigation needs to be performed in order to understand the effect of interface fiber angle on the fracture toughness of CFRP composites.

### 3.5. Prediction Model for Evaluating the Fracture Toughness of CFRP Composites with 0//45 Interface Fiber Angle and Nanomaterials

From the obtained test results, we observed that the change in the interface fiber angle from 0//0 to 0//45 reduced the fracture toughness of the CFRP composites. In addition, using nanomaterials significantly improved the fracture toughness of the CFRP composites, which are necessary to be modeled.

Based on the studies by Zhao et al. [30] and Sou et al. [53], the following simple empirical method was proposed to predict the fracture toughness ($G_{Ic,0//45}$) of the CFRP composites with the interface fiber angle of 0//45 incorporating nanomaterials:

(4) $$G_{Ic,0//45}=G_{Ic,0//0}\left[f_{m}+\lambda V_{f}\right]$$

where $G_{Ic,0//0}$ is the fracture toughness of pure CFRP composites with the interface fiber angle of 0//0, $f_{m}$ is the failure index that expresses the effect of the interface fiber angle, λ is the calibrated factor related to the type of nanomaterials, and $V_{f}$ is the fiber volume fraction of nanomaterials with respect to epoxy resin/hardener mixture. In this study, the fiber volume fractions of MWCNTs, S-MWCNT-COOH, and GnPs were calculated according to Yue et al. [54] and they showed the same value of 1.02 vol.%.

In this study, the fracture toughness ($G_{Ic,0//0}$) of CFRP0-0 with the interface fiber angle of 0//0 is necessary for evaluating $G_{Ic,0//45}$, and it could be evaluated using Equation (2). Notably, $G_{Ic,0//0}$ has the following three parameters: data reduction factor ($A_{1}$), compliance parameter (C), and applied load (*P*). The data reduction factor ($A_{1}$) of CFRP0-0 was proposed to be 30.0 (mm/N)^−1/3^, as presented in Table 4. Subsequently, the compliance parameter (*C*) could be defined using the relationship between delamination length (*a/h*) normalized via thickness and the cube root of compliance (*C*^1/3^) as shown in Equation (5), which was determined using the test results depicted in Figure 10. One has the following:

(5) $$\frac{a}{h}=A_{1}C^{1/3}-5.4$$

**Figure 16:**
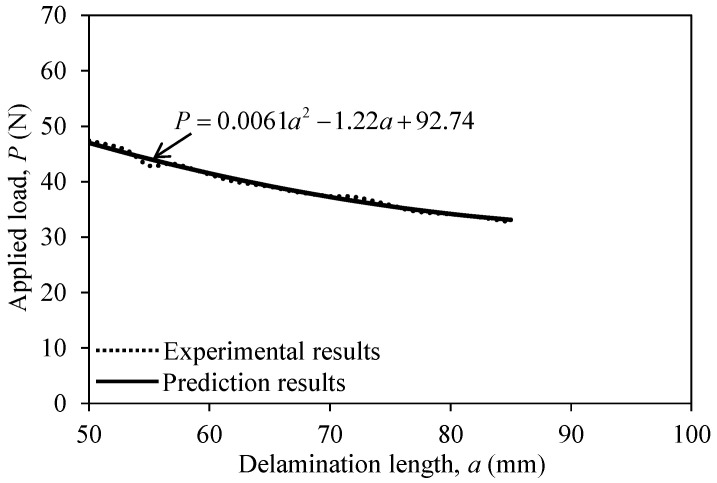
Applied load versus delamination length of the CFRP0-0 specimen.

Based on the experimental results depicted in Figure 16, the applied load (*P*) according to the delamination length (*a*) of the CFRP0-0 specimen could be defined as follows:

(6) $$P=0.0061a^{2}-1.22a+92.74$$

In this study, the failure index (*f_m_*) of the test specimens with the interface fiber angle of 0//45 was proposed to be 0.70 for all the types of nanomaterials. Notably, this value of 0.70 is lower than 1.32, which was suggested by Zhao et al. [30], as the materials and stacking sequences used to fabricate DCB specimens could affect the failure index. Finally, for achieving the best curve fitting, the calibrated factor (*λ*) was empirically determined to be 41, 46, and 52 for S-MWCNT-COOH, MWCNTs, and GnPs, respectively.

**Figure 17:**
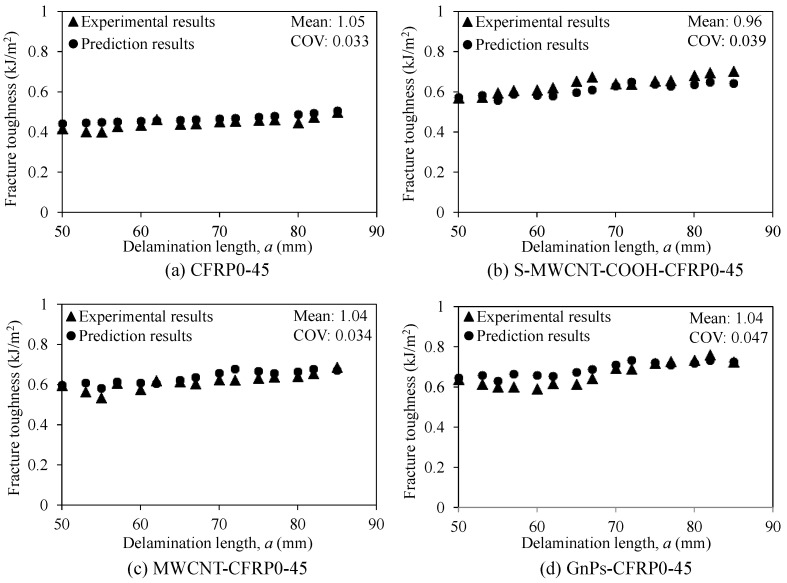
Fracture toughness predicted using the proposed model and evaluated on the basis of experimental results: (**a**) CFRP0-45, (**b**) S-MWCNT-COOH-CFRP0-45, (**c**) MWCNT-CFRP0-45, and (**d**) GnPs-CFRP0-45.

Figure 17 depicts the fracture toughness obtained from the prediction model and experimental results. From the figure, it can be seen that the prediction results were in a good agreement with the experimental results. Both the mean and coefficient of variation (COV) of the ratio of prediction and experimental results were approximately 1.0 and 3.3–4.7%, respectively. Generally, for the limited test data employed in this study, the proposed model could be used to predict the fracture toughness of the DCB specimens with the interface fiber angle of 0//45 incorporating nanomaterials.

## 4. Conclusions

The present study investigated the effects of nanomaterials and interface fiber angle on the fracture behaviors of woven CFRP composites by performing DCB tests. The nanomaterials used were S-MWCNT-COOH, MWCNTs, and GnPs, and the amount of each of them was 1% by weight (1 wt%). Two different series of DCB specimens with different stacking sequences of [0_6_//0_6_] and [(0/45)_3_//(0/45)_3_], respectively, were fabricated and tested. In the first series, only 0° (or 90°) carbon fiber fabrics were used at the delamination surface, while in the second series, 0° (or 90°) and ±45° carbon fiber fabrics were used together. The mode I fracture tests were performed according to ASTM D5528-13, and the fracture toughness was characterized using the MCC method. The primary findings can be summarized as follows:

(1) Using nanomaterials effectively enhanced the flexural rigidities, peak loads, and residual loads of the CFRP composites. For example, in the second series, MWCNT-CFRP0-45 displayed 29.5% higher peak load and 35.9% higher residual load than those of the control specimen CFRP0-0.

(2) The flexural rigidity, peak load, and residual load of each specimen in the test series with the stacking sequence of [0_6_//0_6_] were considerably higher than those of the corresponding specimen in the test series with the stacking sequence of [(0/45)_3_//(0/45)_3_].

(3) The mode I fracture toughness of the test specimens with nanomaterials was higher than that of pure CFRP. Particularly, using GnPs was more effective in enhancing the fracture toughness than using other nanomaterials.

(4) The change in the interface fiber angle from 0//0 to 0//45 could reduce the initiation and propagation fracture toughnesses of the test specimens.

(5) An empirical model was proposed to predict the fracture toughness of the CFRP composites with nanomaterials using on- and off-axis carbon fiber layers together. The prediction results showed a good agreement with the experimental results.

## Figures and Tables

**Table 1:** Geometries and properties of the nanomaterials.

Nanomaterials	Carbon Purity (wt%)	Sizes
Multi-walled carbon nanotubes (MWCNTs)	>95	Outside diameter: (30–50) nm
		Inside diameter: (5–12) nm
		Length: (10–20) μm
		Specific surface area > 60 m^2^/g
		Density: 2.1 g/cm^3^
Graphene nanoplatelets (GnPs)	>95	Average number of layers: 6
		Max. number of layer: 32
		Diameter: (4–12) μm
		Thickness: 2–18 nm
		Specific surface area: 500–1200 m^2^/g
		Density: 2.1 g/cm^3^
COOH-functionalized short multi-walled carbon nanotubes (S-MWCNT-COOH)	>95	Outside diameter: (5–15) nm
		Inside diameter: (3–5) nm
		Length: (0.5–2) μm
		Specific surface area > 233 m^2^/g
		Density: 2.1 g/cm^3^

**Table 2:** Mechanical characteristics of the test specimens.

Specimens	Stacking Sequence	Flexural Rigidity, *K* (N/mm)	SD ^(1)^	Peak Load, *P*_u_ (N)	SD ^(1)^	Residual Load ^(2)^, *P*_R_ (N)	SD ^(1)^
CFRP0-0	[0_6_//0_6_]	5.15	1.21	46.78	2.48	31.95	2.59
S-MWCNT-COOH-CFRP0-0		3.79	1.01	48.74	4.75	35.19	4.17
MWCNT-CFRP0-0		5.39	1.76	45.91	5.82	29.82	5.11
GnPs-CFRP0-0		5.06	1.67	60.58	2.32	43.41	8.49
CFRP0-45	[(0/45)_3_// (0/45)_3_]	2.95	1.00	29.72	2.64	20.06	1.48
S-MWCNT-COOH-CFRP0-45		4.67	1.21	38.54	1.14	23.35	2.39
MWCNT-CFRP0-45		4.10	0.49	39.21	3.47	26.76	3.34
GnPs-CFRP0-45		2.59	0.65	35.98	3.74	25.06	1.43

^(1)^ Standard deviation. ^(2)^ The residual load was determined at the displacement of 40 mm.

**Table 3:** Factors to express the relationship between crack mouth opening displacement and load line displacement of the DCB specimens.

Specimens	Factors			${\Delta_{0}\;}^{\left(1\right)}$ (mm)
	$k_{1}$	$k_{2}$	$k_{3}$	
CFRP0-0	−0.0054	0.89	−9.50	11.48
S-MWCNT-COOH-CFRP0-0	−0.0053	0.90	−12.86	15.97
MWCNT-CFRP0-0	−0.0055	0.87	−10.60	13.30
GnPs-CFRP0-0	−0.0030	0.80	−12.70	16.95
CFRP0-45	−0.0044	0.82	−10.00	13.12
S-MWCNT-COOH-CFRP0-45	−0.0044	0.75	−8.10	11.59
MWCNT-CFRP0-45	−0.0047	0.84	−12.86	16.91
GnPs-CFRP0-45	−0.0037	0.78	−11.00	15.21

^(1)^ Load line displacement when crack propagation begins.

**Table 4:** Compliance parameter experimentally determined using the MCC method.

Specimens	${A_{1,\exp}\;}^{\left(1\right)}$ ((mm/N)^−1/3^)	SD ^(2)^	${A_{1,pro}\;}^{\left(3\right)}$ ((mm/N)^−1/3^)
CFRP0-0	31.2	1.47	30.0
S-MWCNT-COOH-CFRP0-0	30.2	3.75	30.0
MWCNT-CFRP0-0	23.2	3.86	23.0
GnPs-CFRP0-0	37.1	7.43	37.0
CFRP0-45	25.11	4.19	25.0
S-MWCNT-COOH-CFRP0-45	20.8	3.87	20.0
MWCNT-CFRP0-45	25.1	4.43	25.0
GnPs-CFRP0-45	20.0	2.93	20.0

^(1)^ Data reduction factor obtained from the test data in this study. ^(2)^ Standard deviation. ^(3)^ Proposed data reduction factor.

**Table 5:** Mode I fracture toughness of the DCB specimens.

Specimens	Stacking Sequence	Interface Fiber Angle	Amount of Nanomaterials (by Weight, wt%)	$\mathrm{Fracture}\;\mathrm{Toughness},\;G_{Ic}\;(\mathrm{kJ}/m^{2})$			
				Initiation, *G_Ic,in_*	SD ^(1)^	Propagation, *G_Ic,prop_*	SD ^(1)^
CFRP0-0	[0_6_//0_6_]	0//0	0	0.522	0.058	0.587	0.061
S-MWCNT-COOH-CFRP0-0			1	0.650	0.142	0.744	0.192
MWCNT-CFRP0-0				0.647	0.167	0.751	0.158
GnPs-CFRP0-0				0.748	0.156	0.901	0.315
CFRP0-45	[(0/45)_3_// (0/45)_3_]	0//45	0	0.417	0.100	0.500	0.117
S-MWCNT-COOH-CFRP0-45			1	0.571	0.044	0.704	0.112
MWCNT-CFRP0-45				0.596	0.206	0.687	0.023
GnPs-CFRP0-45				0.639	0.143	0.725	0.160

^(1)^ Standard deviation.
